# Stiffness of photocrosslinkable gelatin hydrogel influences nucleus pulposus cell properties*in vitro*

**DOI:** 10.1111/jcmm.16141

**Published:** 2020-12-02

**Authors:** Panpan Xu, Jingjing Guan, Yu Chen, Hui Xiao, Tianhao Yang, Hengheng Sun, Nan Wu, Changchun Zhang, Yingji Mao

**Affiliations:** ^1^ Department of Orthopaedics First Affiliated Hospital Bengbu Medical College Bengbu China; ^2^ Anhui Province Key Laboratory of Tissue Transplantation Bengbu Medical College Bengbu China; ^3^ School of Life Sciences Bengbu Medical College Bengbu China; ^4^ Department of Plastic Surgery The First Affiliated Hospital of Bengbu Medical College Bengbu China

**Keywords:** gelatin methacrylate, microenvironment, nucleus pulposus cells, tissue engineering

## Abstract

A key early sign of degenerative disc disease (DDD) is the loss of nucleus pulposus (NP) cells (NPCs). Accordingly, NPC transplantation is a treatment strategy for intervertebral disc (IVD) degeneration. However, in advanced DDD, due to structural damage of the IVD and scaffold mechanical properties, the transplanted cells are less viable and secrete less extracellular matrix, and thus, are unable to efficiently promote NP regeneration. In this study, we evaluated the encapsulation of NPCs in a photosensitive hydrogel made of collagen hydrolysate gelatin and methacrylate (GelMA) to improve NP regeneration. By adjusting the concentration of GelMA, we prepared hydrogels with different mechanical properties. After examining the mechanical properties, cell compatibility and tissue engineering indices of the GelMA‐based hydrogels, we determined the optimal hydrogel concentration of the NPC‐encapsulating GelMA hydrogel for NP regeneration as 5%. NPCs effectively combined with GelMA and proliferated. As the concentration of the GelMA hydrogel increased, the survival, proliferation and matrix deposition of the encapsulated NPCs gradually decreased, which is the opposite of NPCs grown on the surface of the hydrogel. The controllability of the GelMA hydrogels suggests that these NPC‐encapsulating hydrogels are promising candidates to aid in NP tissue engineering and repairing endogenous NPCs.

## 1 INTRODUCTION

Degenerative disc disease (DDD) is a major cause of neck pain, low back pain and disability in elderly individuals that is detrimental to their quality of life and ability to work.^1^ Recently, with the ageing of the population, DDD has become a major public health issue and economic burden.^2^ Current treatment strategies, including conservative and surgical treatments, do not resolve the underlying mechanisms and can cause complications.^3^ Therefore, there is an urgent need to develop more effective treatments for DDD that address the causal mechanisms.

The intervertebral disc (IVD) has a typical multi‐scale structural organization, consisting of three components: the highly hydrated gelatinous nucleus pulposus (NP) in the centre, the surrounding elastic annulus fibrosus (AF), and the upper and lower cartilaginous endplates.^4^ The NP plays an important role in the axial loading of the spine, as it can distribute hydraulic pressure evenly between adjacent vertebral bodies.^5^ However, in DDD, the NP is in a dehydrated state and cannot achieve fluid pressurization, which increases the axial compression of the NP and AF, resulting in an uneven stress distribution on the IVD and the destruction of the surrounding AF. NP changes are early signs of DDD and include a decreased cell count in the NP, inflammation, reduced synthesis of extracellular matrix (ECM) components (such as type II collagen [Col II] and proteoglycans), and fibrosis.^6^, ^7^ After the onset of DDD, due to the lack of vascular tissue, NP cells (NPCs) suffer nutrient deficiency and difficulty in regeneration, thus limiting endogenous repair.^8^

Tissue engineering and regenerative medicine have provided solutions to this problem. Hydrogels are considered the most promising candidate for NP tissue engineering because they have similar characteristics and functions to those of natural NP tissues, such as a high water content and fluid pressurization effect.^9^, ^10^ Many types of hydrogels have been evaluated for NP tissue engineering, including alginate,^11^, ^12^ fibrin/fibrinogen,^13^, ^14^ collagen,^15^, ^16^ carboxymethyl cellulose,^17^, ^18^ gellan gum,^19^, ^20^ chitosan,^21^, ^22^ and composite hydrogels.^23^, ^24^ Ideally, these self‐healing hydrogels should have certain biological properties, such as an appropriate gel fraction, injectability, the ability to support encapsulated cell proliferation and ECM secretion, high mechanical strength, degradability, biocompatibility, and no side effects after implantation.^25^ More importantly, in advanced DDD, due to the damaged IVD structure, the NP will bear a greater axial compression force, and appropriate mechanical properties will help maintain the 3D structure of the implanted NP at an early stage.^26^ Furthermore, the hydrogel must facilitate the survival, growth, and migration of NPCs. Most hydrogels developed to date do not fulfil these criteria; therefore, they cannot support the long‐term regeneration of the NP.

Gelatin methacrylate (GelMA) is a photosensitive hydrogel that not only retains the unique properties of gelatin but can also photocoagulate from liquid to solid via chemical cross‐linking methacrylamide groups.^27^ With the right photoinitiator, the hydrogel can be cross‐linked to form a solid within a few minutes or even a few seconds, thereby minimizing cytotoxicity.^28^ Moreover, since the GelMA hydrogel is transparent, it is easy to observe cells that are encapsulated or those on the surface. Notably, the mechanical properties, degradability, and biological properties of the GelMA hydrogel can be modified by altering the ratio of methacrylamide or the photopolymerization time,^27^, ^29^ making it possible to precisely control its performance. The 3D GelMA hydrogel system has characteristics similar to those of natural NP tissues, including a high water content and porous structure, thereby facilitating the penetration of nutrients and oxygen as well as providing a good local microenvironment for growing NPCs.^30^ GelMA hydrogel scaffolds have been utilized for bone repair, cardiovascular disease, and skin regeneration.^31^, ^32^, ^33^ However, their application to NP tissue engineering has yet to be explored.

In this study, we used GelMA hydrogel for NPC transplantation to support NP regeneration. To determine the optimal hydrogel concentration for NP regeneration, we adjusted the concentration of GelMA hydrogel, thus modifying its mechanical properties and cell compatibility in a controlled manner. We first evaluated the mechanical properties of GelMA hydrogels at different concentrations. Following this, we examined their microstructures and hydration properties. Finally, we assessed the ability of NPCs to survive, extend, and deposit ECM in the 3D GelMA hydrogel system.

## 2 MATERIALS AND METHODS

### 2.1 Preparation and characterization of GelMA hydrogels

**FIGURE 1:**
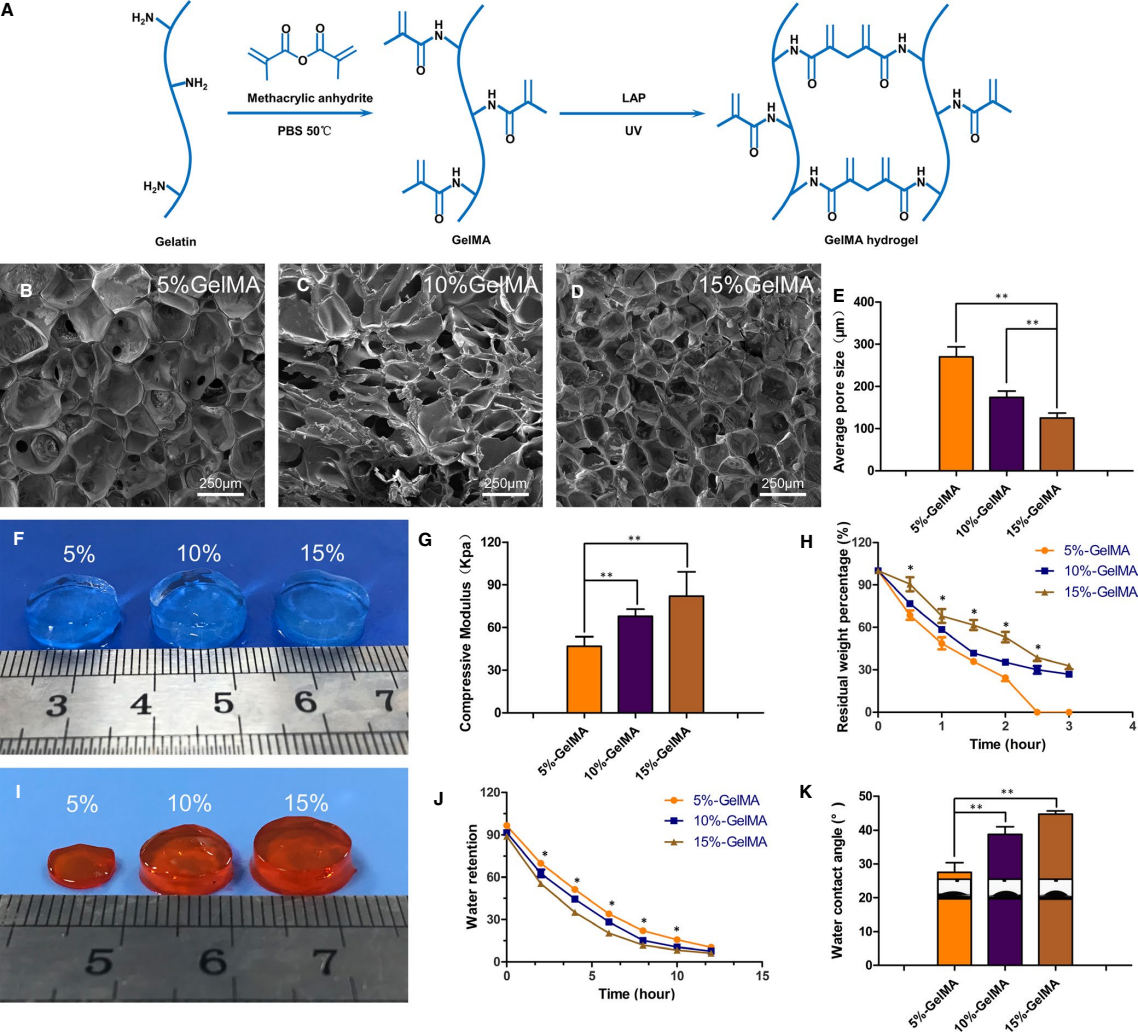
Characterization of GelMA hydrogels. (A) Schematic representation of GelMA hydrogel formation by the methacrylic anhydride and gelatin reaction. (B‐D) SEM images of GelMA hydrogel with three concentrations after freeze‐drying. (E) Average pore size of hydrogels. (F) Appearance of hydrogels at three concentrations. (G) Compression modulus of hydrogels at different concentrations. (H) Degradation of GelMA hydrogel at different concentrations in vitro. (I) In vitro appearance of hydrogel degradation at 2 h. (J) Swelling property of hydrogel. (K) WCA of hydrogel. (*P < .05, **P < .01)

According to the literature,^27^, ^34^ gelatin (Suzhou Intelligent Manufacturing Research Institute, China) was dissolved in a preheated phosphate buffer solution (PBS; 50°C). Methacrylate anhydride (Suzhou Intelligent Manufacturing Research Institute, China) was added to the gelatin solution at a rate of 0.5 mL/min and allowed to react for 2 hours. The mixture was packed into dialysis bags (Mw: 8000‐14 000) to remove unbound methacrylic acid and other small molecular impurities. After a week of continuous dialysis, replacing the deionized water twice daily, the dialysate was heated to 60°C and then filtered using a 0.22 μm filtration membrane. The filtrate was then freeze‐dried to a white foam and collected for use. Thereafter, 5%, 10%, and 15% GelMA (w/v) were dissolved with 0.05% of the photoinitiator lithium aryl phosphonate in PBS, and a hydrogel unit was prepared by irradiating it under a UV light for 12 seconds (Figure 1A).

#### 2.1.1 Aperture size evaluation

The three hydrogels with different concentrations were frozen at −80°C overnight and then freeze‐dried at −40°C. The sample surface was sputter‐coated with gold (SC7620, Quorum Technologies, UK) and then observed and photographed using a scanning electron microscope (SEM; Hitachi, Kyoto, Japan). The average pore sizes of the samples were measured using ImageJ software (National Institute of Health, Bethesda, MD, USA).

#### 2.1.2 Hydrogel swelling analysis and water contact angles

After freeze‐drying, the weight of the hydrogel was recorded as W_0_, and the weight of hydrogel soaked in PBS overnight was recorded as W_1_. Following this, the scaffold was maintained at room temperature (25℃). According to different time points, the weight of the hydrogel was recorded as W_t_ after water was sucked off its surface. The percentage of water retention (W_R_) at the different periods was recorded using the following equation: W_R_ = (W_t_‐W_0_) /W_1_ × 100%.

The wettability of hydrogels was assessed at 25°C via the water contact angle (WCA) and droplet shape. 2 U/mL of collagenase II at 37ºC.

#### 2.1.3 Hydrogel degradation

The weight of the freeze‐dried hydrogel was denoted as W_0_. The hydrogel was then soaked in 2 U/mL of collagenase II (Sigma‐Aldrich, USA) in a 37°C, constant temperature water bath. When taken out at different time points, the weight after freeze‐drying was recorded as W_t_. The percent degradation rate (W_D_) of the sample was denoted as W_D_ = W_t_/W_0_ × 100%.

#### 2.1.4 Mechanical testing

Hydrogel scaffolds with a diameter of 8 mm were prepared and then immersed in PBS overnight. The compression modulus of the hydrogels of different concentrations was determined using mechanical testing instruments (Shanghai Heng Yi Precision Instrument Co. Ltd., China) at a 20% strain rate.

### 2.2 Isolation and culture of NPCs

All animal feeding and surgical procedures were approved by the Animal Ethics Committee of Bengbu Medical College. According to the literature,^35^, ^36^ healthy Sprague‐Dawley (SD) rats (200‐230 g) were killed via an intraperitoneal injection of pentobarbital sodium. The intervertebral discs in the tail were collected, and subsequently, the NP tissue was separated, cut into pieces under sterile conditions, and then treated with 0.2% collagenase II in an incubator at 37°C for 1 hour. After washing and centrifuging, NPCs were cultured in DMEM/F12 (Gibco, USA) complete medium containing 10% foetal bovine serum (Hyclone, China) and 100 U/mL penicillin‐streptomycin (Invitrogen, USA). The cells were cultured at 37°C in 5% CO_2_. The complete medium was replaced every two days and the cells were used at the third passage.

### 2.3 NPC adhesion

The GelMA hydrogel was injected into a 96‐well plate, irradiated under ultraviolet light, and plated with 2 × 10^3^ NPCs in each well. The cultures were washed with PBS after 4 and 7 days, the cells were fixed with 4% paraformaldehyde (Sigma, USA) for 20 minutes, and then infiltrated with 0.5% Triton X‐100 (Invitrogen) pairs of cells for 20 minutes. FITC‐phalloidin (Invitrogen, USA) was incubated at room temperature and in the dark for 40 minutes. After washing with PBS, the nuclei were stained with DAPI (Invitrogen). The actin filaments and nuclei of NPCs on hydrogels were observed under an inverted microscope (Olympus, Japan).

### 2.4 NPCs encapsulated in hydrogels

A total of 5 × 10^6^ cells/ml NPCs were suspended and mixed with the GelMA hydrogel. Then, the suspension was injected into a circular mould with a diameter of 5 mm and a height of 2 mm, and the cell‐encapsulated hydrogel was prepared using ultraviolet irradiation. The cell‐encapsulated hydrogel scaffold was immediately placed in a complete medium and then cultured in a humidified incubator at 37°C and 5% CO_2_. The medium was changed every other day.

### 2.5 Live/dead assay and cell proliferation in the hydrogels

After 7 days of in vitro culture, viability of the GelMA‐encapsulated cells at different concentrations was assessed. The samples were washed with PBS and then incubated with the Calcein‐AM/PI double stain kit (Invitrogen) at 37°C and 5% CO_2_ for 40 minutes. After washing with PBS, the samples were observed using laser scanning confocal microscopy (LSCM; Olympus, Japan).

Proliferation of the NPCs in the hydrogel was measured using the cell counting kit‐8 (CCK8, Invitrogen). The medium was removed at each time point, and 10% CCK‐8 was added to the fresh medium and incubated for 3 hours. Thereafter, the incubation solution was transferred to a 96‐well plate, and the absorbance of the solution was measured at 450 nm with a micro tablet reader (Invitrogen).

### 2.6 Immunofluorescence analysis

The samples were washed with PBS and fixed with 4% paraformaldehyde at 4°C for 30 minutes. The scaffolds were permeated with 0.5% Triton X‐100 for 30 minutes, sealed with 5% BSA (Invitrogen) for 1 hour, and then incubated with the primary antibodies anti‐aggrecan (Affinity Biosciences, USA) and anti‐Col II (Affinity Biosciences, USA) at 4°C overnight. Next, the primary antibodies were collected and the scaffolds were washed with PBS. Next, the scaffolds were incubated with Alexa Fluor 488‐conjugated Goat anti‐rabbit (Proteintech, USA) and Cy3‐conjugated Goat anti‐rabbit (Proteintech, USA) for 1 hour. Finally, the scaffolds were stained with DAPI for 15 minutes and were observed and photographed using LSCM.

### 2.7 Histological analysis

Histological analysis was performed after 7 and 14 days of cell encapsulation. The samples were washed with PBS and fixed with 4% paraformaldehyde for 1 day. Then OCT (Sakura Finetek, Japan) and ethanol were used to dehydrate 5% and 10%‐15% of the samples, respectively. The samples were embedded with OCT and cut into 9 μm‐thick slices in a cryogenically frozen slicer (Leica, Germany). The samples were stained in the following order: haematoxylin and eosin (H&E), Masson and toluidine blue. The sections were observed using an inverted microscope.

### 2.8 Western blotting analysis

The hydrogel‐encapsulated scaffolds were repeatedly suspended in the GelMA lysate (Suzhou Intelligent Manufacturing Research Institute, China) for 1 hour until the GelMA dissolved completely. After that, the protein was extracted and analysed using Western blotting with primary antibodies (anti‐aggrecan and anti‐Col II) and the secondary antibody HRP‐conjugated goat anti‐rabbit (Proteintech, USA). Finally, Image J software was used to measure the density of the immunoreactive bands, and quantify and normalize them.

### 2.9 Statistical analysis

All experimental data are presented as mean ± standard deviation (SD) and were statistically analysed using ANOVA with GraphPad Prism 5.00 software (La Jolla, CA, USA).

## 3 RESULTS

### 3.1 Characteristics of the GelMA hydrogel

Pore size is attributed to the cross‐linking distance within the GelMA hydrogel that is mainly affected by the cross‐link density. As determined using SEM, all cross‐sections of the GelMA hydrogels at different concentrations showed a porous honeycomb structure, which can help to facilitate nutrient transport and provide space for proliferating and expanding encapsulated cells (Figure 1B–D). Average pore sizes for 5%, 10%, and 15% (w/v) GelMA hydrogels were 270.0 ± 10.51 μm, 174.2 ± 6.47 μm, and 124.8 ± 5.26 μm, respectively, indicating that pore size decreased as the hydrogel concentration increased (Figure 1E).

The elasticity of the GelMA hydrogel and resistance to deformation are critical parameters for the long‐term structural stability of the NP. To determine the relationship between the concentration of the GelMA hydrogel and mechanical strength, we performed compression tests using 5%, 10%, and 15% (w/v) GelMA hydrogels. The compression modulus was the lowest for the 5% (46.78 kPa) hydrogel and the highest for the 15% (82.05 kPa) hydrogel. Therefore, there was a positive correlation between the hydrogel concentration and compression modulus (Figure 1G).

Biodegradability is a key characteristic of hydrogel used in tissue engineering. As shown in Figure 1H–I, the degradation rate decreased gradually as the concentration of the hydrogel increased. At 2.5 hours, the 5% GelMA hydrogel completely dissolved (Figure 1I), while the 10% and 15% GelMA hydrogels reduced in volume, with degradation rates of 30% and 38.6%, respectively (Figure 1H). This difference might be explained by weaker cross‐linking between methacrylamides in the low‐concentration GelMA hydrogel, which makes more significant spaces among the bonding molecules. Under the influence of the weak bond between methacrylate groups, degradation is faster. The main driving force of degradation is related to the excessively low substrate gelatin.

As high water content can facilitate the transport of nutrients, the W_R_ capacity and WCA are important parameters. In our experiment, the W_R_ capacity of the 5% GelMA hydrogel was consistently higher than those of the 10% and 15% GelMA hydrogels. This may be due to the low cross‐link density of the 5% GelMA hydrogel, which enabled more water to penetrate (Figure 1J). The WCA for the 5%, 10%, and 15% GelMA hydrogels were 27.50 ± 1.44°, 38.75 ± 1.11°, and 44.75 ± 0.48°, respectively (Figure 1K). Therefore, the 5% GelMA hydrogel had the highest hydrophilicity and wettability, that is, parameters that are the most useful in facilitating cell survival.

### 3.2 Cell adhesion to the surface of the 2D GelMA hydrogel

**FIGURE 2:**
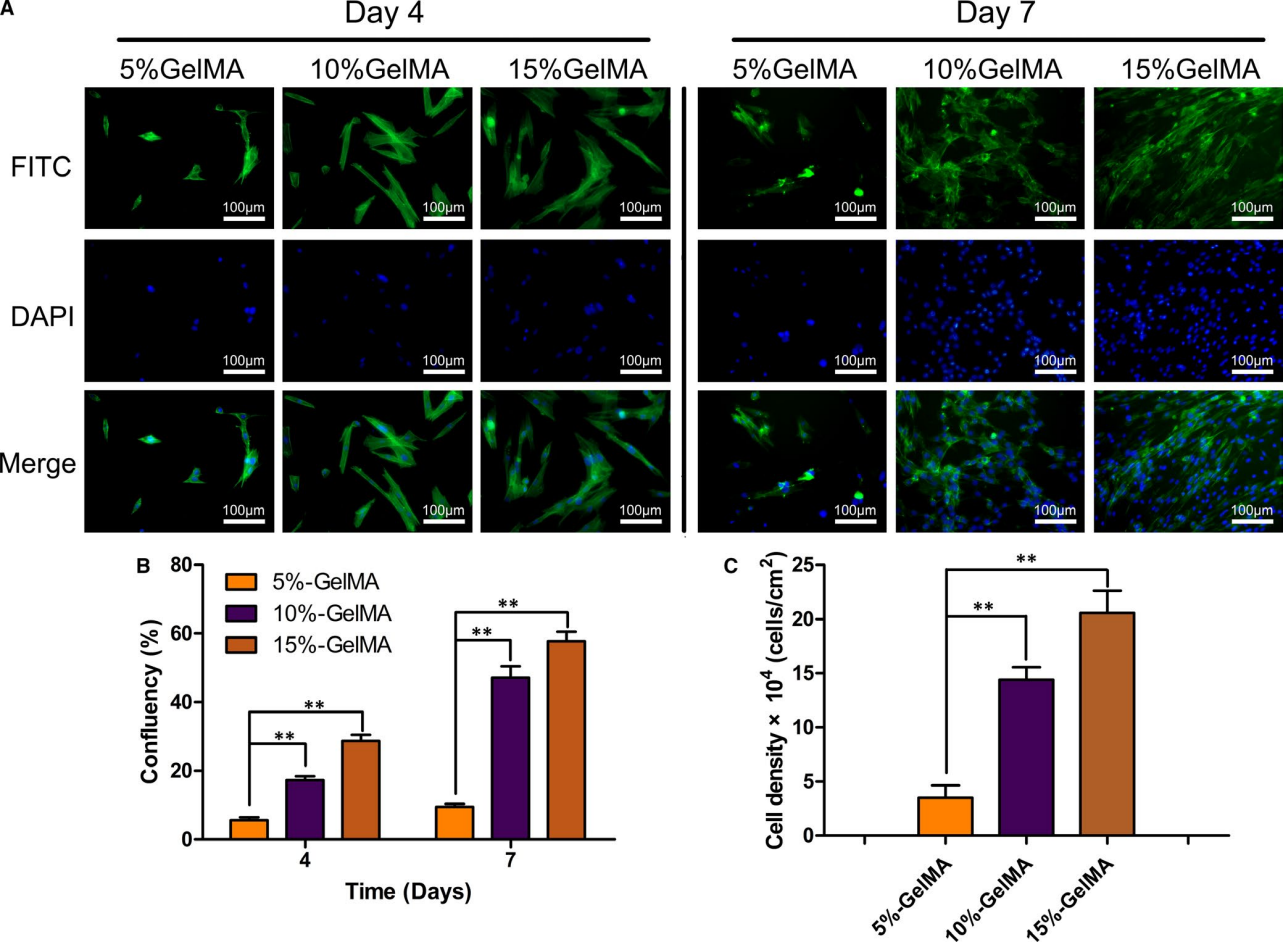
Adhesion, proliferation and migration of NPCs on the GelMA hydrogel surface. (A) FITC‐labelled phalloidin /DAPI was used to stain actin filaments and nuclei of NPCs cultured for 4 and 7 days. (B) Over time NPCs’ confluence on GelMA hydrogel with different concentrations showed significant differences. (C) Cell density on the hydrogel surface on day 7 was measured and calculated by the number of DAPI staining nuclei positive in the restricted area. (*P < .05, **P < .01)

Cell‐scaffold adhesion is critical for the survival and growth of cells. We found that NPCs had stronger adhesion to the surface of the 15% GelMA hydrogel than to the surface of the 5% and 10% GelMA hydrogels (Figure 2). At 4 days after cell transplantation, the NPCs adhered to the surfaces of the GelMA hydrogels at all concentrations. Based on confluence (ie the percentage of adherent cells), NPC extension and migration were the greatest on the surfaces of the 10% and 15% GelMA hydrogels, whereas cells on the 5% GelMA hydrogel were mostly round and had no extension (Figure 2A). After 7 days, compared with cells on the 10% and 15% GelMA hydrogels, cells on the 5% GelMA hydrogel showed extension but still showed less confluence (Figure 2A). Moreover, confluence increased substantially on the 10% and 15% GelMA hydrogels, with values two‐ to three‐fold higher than those at 4 days (Figure 2B). Confluence on the 5% GelMA hydrogel did not show a considerable change over time and was significantly different from that of the 10% and 15% GelMA hydrogels (*P* < .01). The cell density (based on nuclear DAPI staining in a target area) after 7 days also showed significant differences concerning hydrogel concentration, consistent with the results for confluence (*P* < .01; Figure 2C).

For overall NPC morphology, the 5% GelMA hydrogel showed cell aggregation and expansion, and the degree of confluence increased as the concentration of the hydrogel increased. The relationship between confluence and concentration of hydrogel was not simply a reflection of increases in extension. The difference in morphology was also related to increases in cell quantity and aggregation. This difference may be due to the increased hardness of the hydrogel with an increase in the hydrogel concentration, resulting in a denser structure and improved bioactivity of the seeded surface cells.

### 3.3 Cell encapsulation in the 3D GelMA hydrogel

**FIGURE 3:**
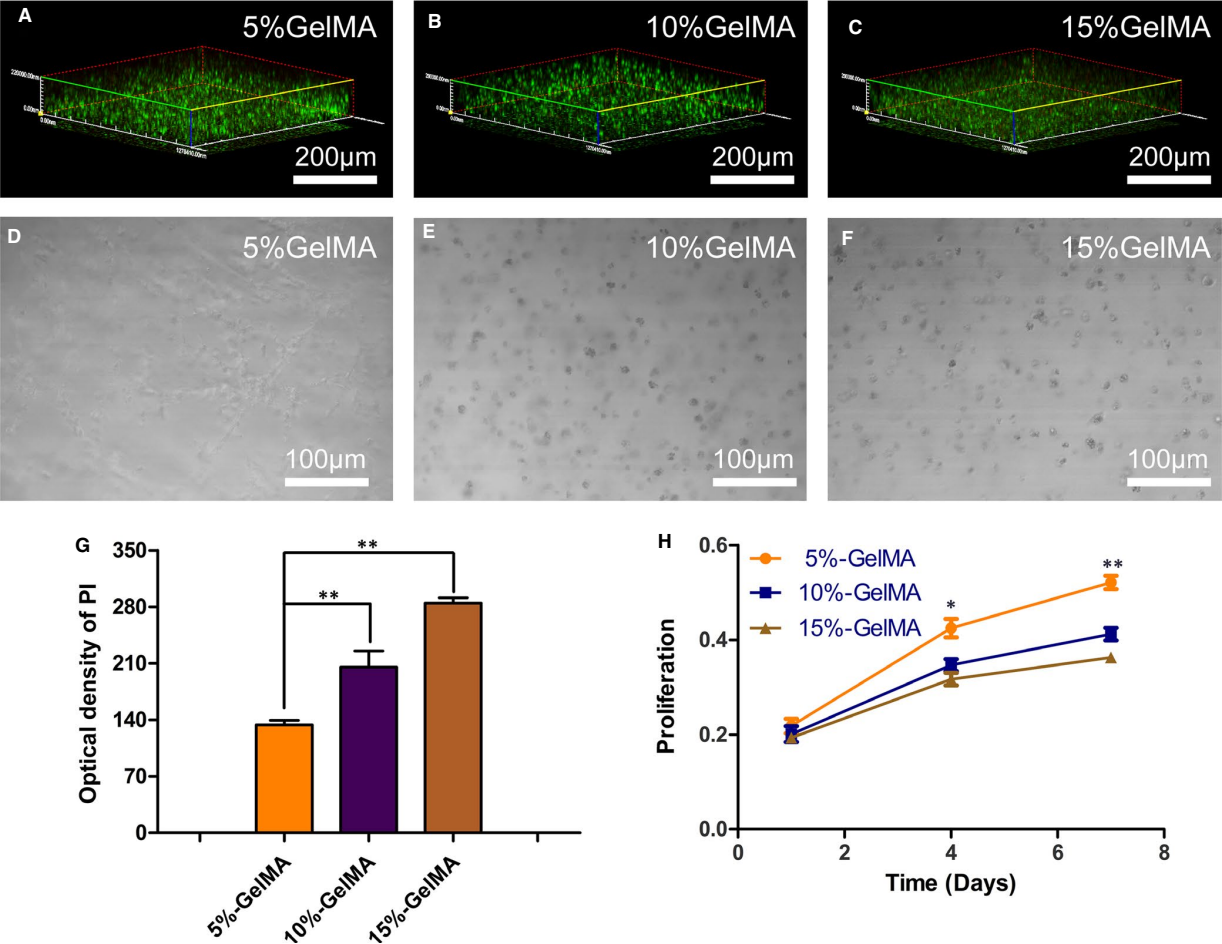
Cytocompatibility of NPCs and GelMA hydrogel. (A‐C) Live/dead staining analysis of NPCs encapsulated in GelMA hydrogel on day 7, Calcein‐AM (green) to indicate live cells and PI (red) to indicate dead cells. (D‐F) After 7 days of culture, 3D images showed that the elongation length of the cells was inversely proportional to GelMA hydrogel concentration. (G) Quantitative analysis of dead cells in the hydrogel. (H) Cell proliferation of NPCs encapsulated in GelMA hydrogel at days 1, 4 and 7, respectively (*P < .05, **P < .01)

To detect the proportion of live cells, live/dead cell staining was performed 7 days after the encapsulation of NPCs in the GelMA hydrogel (Figure 3A–C). As visualized using fluorescence imaging, most of the cells encapsulated in the hydrogels were alive, and almost no dead cells were detected for all three concentrations; the proportion of dead cells was the lowest in the 5% GelMA hydrogel (*P* < .01; Figure 3G). These results indicate that NPCs had high viability on 7 days after encapsulation, and the hydrogels had no cell toxicity and exhibited good cell compatibility. In the 5% GelMA hydrogel, NPCs grew outward, migrated, and connected with the surrounding cells (Figure 3D). However, in the 10% and 15% GelMA hydrogels, cells maintained a rounded shape (Figure 3E–F).

To determine whether the three concentrations of hydrogels have a positive effect on the proliferation of NPCs, CCK‐8 assays were conducted after 1, 4, and 7 days (Figure 3H). After 1 day, there was no difference in cell growth rates among the three concentrations of hydrogels. In contrast, after 4 and 7 days, the proliferation of encapsulated NPCs significantly decreased at the higher hydrogel concentrations, and the difference was the highest after 7 days (*P* < .01).

**FIGURE 4:**
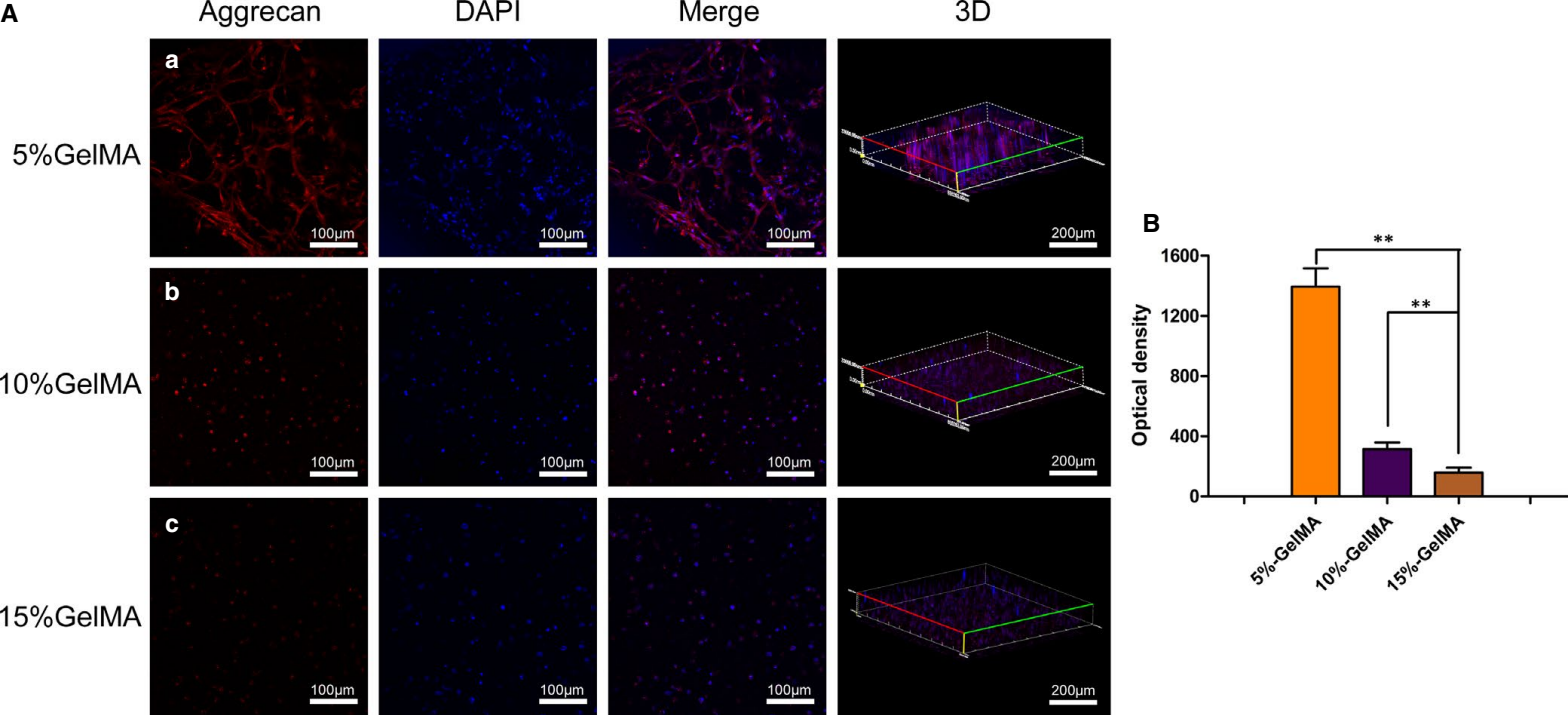
Expression of aggrecan of NPCs encapsulated in the GelMA hydrogel. (A) Immunofluorescence staining of NPCs 7 days after encapsulation at different concentrations GelMA hydrogels. (B) Quantitative optical density analysis of aggrecan expression. (*P < .05, **P < .01)

**FIGURE 5:**
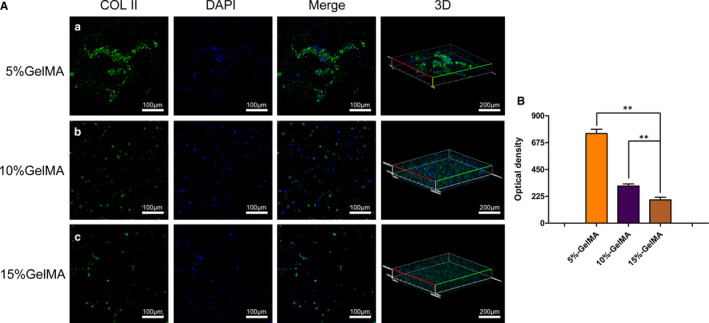
Expression of Col II of NPCs encapsulated in GelMA hydrogel. (A) Immunofluorescence staining of NPCs 7 days after encapsulation at different concentrations GelMA hydrogels. (B) Quantitative optical density analysis of Col II expression. (*P < .05, **P < .01)

Aggrecan and Col II are important components of the NP. Therefore, immunofluorescence was used to evaluate the growth and protein expression in the encapsulated NPCs after 7 days (Figures 4, 5). There was an extensive mesh structure in the 5% GelMA hydrogel, consisting mainly of NPCs connected through the pores within the hydrogel. In contrast, cells in the 10% and 15% GelMA hydrogels remained rounded (Figure 4A). Based on quantitative measurements of the optical density, we found that the expression levels of aggrecan were significantly higher when cells were embedded in the 5% GelMA hydrogel than in the 10% and 15% GelMA hydrogels (*P* < .01; Figure 4B).

We also examined the expression of Col II. Compared with the densities of cells encapsulated in the 5% GelMA hydrogel, those of cells encapsulated in the 10% and 15% GelMA hydrogels were significantly lower, with no extension (Figure 5A). Based on optical densities, Col II expression patterns were consistent with those of aggrecan, with the highest expression levels in NPCs cultured within the 5% GelMA hydrogel (*P* < .01; Figure 5B).

### 3.4 Histological analysis

**FIGURE 6:**
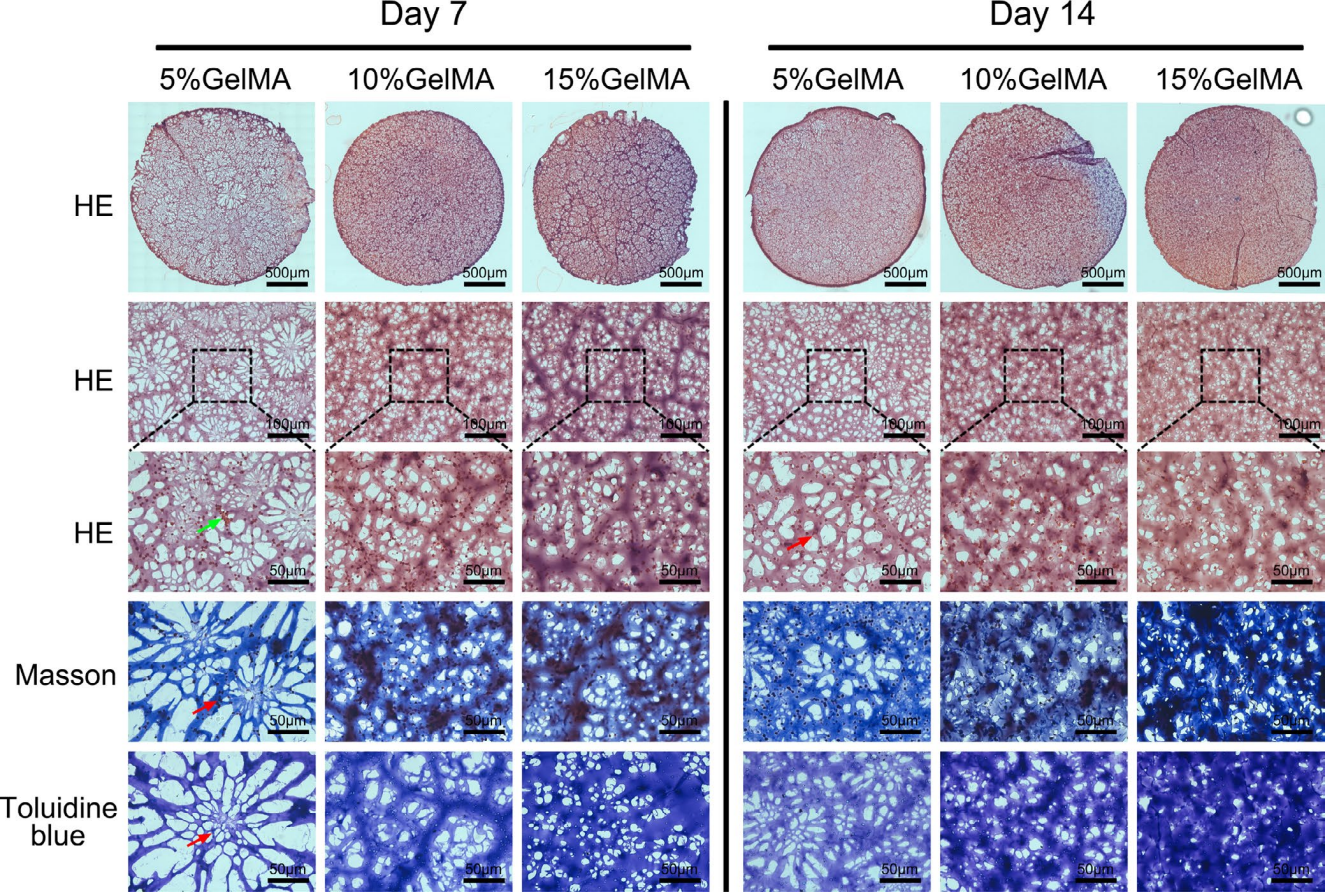
Histological observation of GelMA hydrogel‐encapsulated NPCs. Micrographs showed H&E, Masson and toluidine blue staining of NPCs encapsulated in 5%, 10% and 15% (w/v) GelMA hydrogel on days 7 and 14 (green arrows indicating cell clusters; the red arrow indicates the single cell)

To observe the histological characteristics of NPCs encapsulated within the GelMA hydrogel, H&E staining was performed on sections of the hydrogels after cells were cultured for 7 and 14 days (Figure 6). In the 5% GelMA hydrogel, changes in the number of cells were observed after 7 days, along with the formation of some cell clusters (Figure 6; green arrows). Moreover, evenly dispersed cells were observed after 14 days (red arrow), indicating that NP‐like cell tissues formed in the 5% GelMA hydrogel. In contrast, NPCs encapsulated in the 10% and 15% GelMA hydrogels did not change significantly during the culture period, and no cell clusters formed. These histological results suggest that the 5% GelMA hydrogel enabled cell survival and the formation of NP‐like cells.

The mesh structure of the hydrogel scaffold was also observed in the H&E sections. The 5% GelMA hydrogel exhibited a sparse, highly porous mesh structure (Figure 6). In contrast, the 10% GelMA hydrogel exhibited a uniform structure with small pores, whereas the 15% GelMA hydrogel had thick, cord‐like matrices and a dense, mesh‐like overall structure.

### 3.5 ECM deposition in hydrogels

Masson trichrome and toluidine blue were used to detect the accumulation of Col II and aggrecan in the three hydrogels after culturing embedded NPCs for 7 and 14 days (Figure 6). After 7 days, the 5% and 10% GelMA hydrogels had more Col II and aggrecan deposits around the encapsulated cells than the 15% GelMA hydrogel, and their expression levels were the highest in NPCs cultured within the 5% GelMA hydrogel. Similarly, in the 5% GelMA hydrogel, a large number of proteoglycans could be observed, with dark staining around the encapsulated cells. After 14 days, the cells encapsulated within the 5% GelMA hydrogel secreted significantly higher amounts of Col II and aggrecan compared to NPCs embedded within the 10% and 15% GelMA hydrogels, as indicated by the sparse ECM.

**FIGURE 7:**
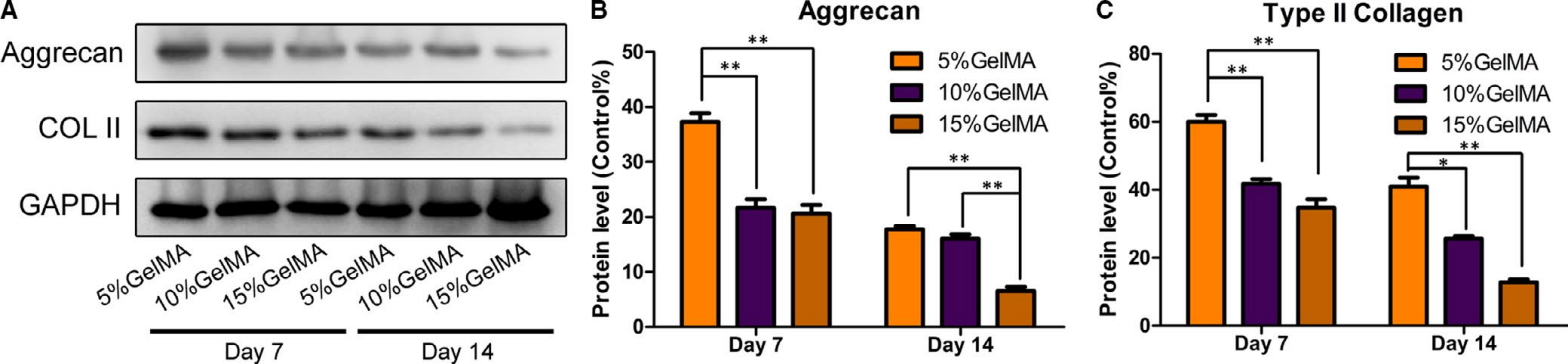
(A) Expression levels of aggrecan and Col II in each group were detected by Western blot. GAPDH protein was used as the control. (B‐C) Aggrecan and Col II quantitative analysis results (*P < .05, **P < .01)

We quantified the expression of the ECM‐related proteins Col II and aggrecan in the hydrogels using Western blotting (Figure 7A). After 7 and 14 days, Col II and aggrecan expression levels were higher for NPCs encapsulated in the 5% GelMA hydrogel than those from the 10% and 15% GelMA hydrogels (Figure 7B–C). In addition, the expression levels of Col II and aggrecan for cells from all of the hydrogel concentrations decreased after 14 days; however, the expression levels were still significantly higher for cells from the 5% GelMA hydrogel than cells from the 10% and 15% GelMA hydrogels (Figure 7B–C; *P* < .05).

## 4 DISCUSSION

In this study, we generated a NPC‐encapsulating 3D hydrogel scaffold with adjustable mechanical properties. At different concentrations, the photosensitive GelMA hydrogel scaffold demonstrated different patterns of cell growth and ECM deposition. The 5% GelMA (w/v) hydrogel was able to support cell survival and proliferation. The expression levels of NPC‐specific ECM proteins, aggrecan and Col II, increased gradually overtime during the culture period, indicating that the 5% GelMA hydrogel could be a suitable scaffold for NP regeneration. Therefore, by adjusting the concentration of the GelMA hydrogel, we optimized its mechanical and biological properties for NP regeneration.

A healthy NP is composed of soft and flexible tissues that resist axial pressure under the AF. The fluid pressurization effect of the NP allows it to resist high buffered pressures.^37^ When the AF breaks and the NP protrudes, the IVD structure is destroyed, making it unable to resist the pressure; this is a common problem in advanced DDD. Therefore, the toughness and hydration properties of scaffolds are important for the retention of their shape and position after transplantation and the long‐term regeneration of the NP.^26^ As a hydrolysis product of collagen, gelatin has been used for a long time in the fields of food science, nucleic acid research, medicine, and tissue engineering.^38^, ^39^, ^40^, ^41^ Since gelatin contains the arginine‐glycine‐aspartate adhesion domain and matrix metalloproteinase (MMP)‐sensitive sites, it can bind to different types of growth factors and promote the proliferation and expansion of different cells types within scaffolds.^42^, ^43^, ^44^, ^45^ The chemical bonds in gelatin can be covalently modified with cytokines to further enhance cell viability and function. Therefore, GelMA hydrogels, composed mainly of denatured collagen, can be combined with different cells and are widely applicable in tissue engineering.

GelMA hydrogels formed by photocrosslinking gelatin and methacrylate can easily form a scaffold under ultraviolet light. GelMA hydrogels with different mechanical properties can be obtained in a controllable manner to ensure good biocompatibility. The mechanical properties of GelMA hydrogels can have a significant effect on cell survival, proliferation, and differentiation.^46^ In our previous studies, we changed the mechanical properties of the GelMA hydrogel to affect the differentiation of co‐cultured neural stem cells and bone marrow mesenchymal stem cells.^47^ In this study, we prepared GelMA hydrogels at three concentrations, 5%, 10%, and 15%, by adjusting the ratio of methacrylate. By modulating the mechanical properties of the GelMA hydrogel, we simulated the natural microstructure of the NP in the IVD. The 5% GelMA hydrogel had a softness similar to that of the NP. It also had good viscoelastic, which confers a fluid pressurization effect, thereby preventing the protrusion of the NP from the IVD. Moreover, the load‐bearing capacity of the NP depends on the internal hydrostatic pressure rather than its viscoelasticity.^48^ The balance between the external pressure and innate hydration properties of the NP plays an important role in maintaining the IVD structure. The hydration properties of hydrogels are the main determinants of the mechanical properties of the NP. Under a high axial load, the hydration properties of the 5% GelMA hydrogel were similar to those of natural NP tissues, suggesting that this hydrogel had compatible load‐bearing properties to withstand transplantation.

The ability of cells to grow, migrate, and connect with adjacent cells in a 3D structure is crucial for applications in NP tissue engineering. Cell‐encapsulating hydrogels such as hyaluronic acid and polyethylene glycol, can ensure even cell dispersal. However, these polymer‐encapsulated cells cannot generally change the surrounding microenvironment, thereby limiting their application in tissue engineering.^49^ Binding motifs can be added by combining the hydrogel with natural ECM or cell adhesion peptides to promote cell adhesion and growth.^49^, ^50^ In general, most binding motifs of hydrogels only connect with connexins or the ends of substrates. However, the binding sites within the GelMA hydrogels are distributed across all chains of the hydrogel, which greatly improves the binding ability of cells. Cells not only adhere to the surface of the GelMA hydrogel but also migrate and expand when encapsulated in a 3D hydrogel system. Moreover, NPC aggregation is an important property that facilitates the formation of cartilage and the maturation of engineered tissues.^51^, ^52^ A high‐density hydrogel structure will obstruct cell aggregation, as the cells are limited by the high‐density mesh and cannot expand.^53^ In this study, the 5% GelMA hydrogel had a low cross‐linking density and large mesh size, and its mechanical and hydration properties were very similar to those of natural NP tissues. Thus, the encapsulated cells aggregated and formed cell clusters. It is possible that the extensive binding sites within the GelMA hydrogel can increase cell adhesion at early stages, and its highly porous structure can facilitate cell growth, migration, and connection at later stages. In contrast, aggregated cell clusters did not form in the 15% GelMA hydrogel, and cell growth was poor after 14 days; thus, the hydrogel could not support the formation of NP tissues.

A good 3D microenvironment increases the viability of NPCs and improves the spatial distribution of ECM components.^15^ The low‐density 5% GelMA hydrogel had a significantly higher percentage of viable cells after 7 days than the 10% and 15% GelMA hydrogels. ECM deposition also increased gradually with time. In contrast, the small mesh size of the hydrogels with high cross‐linking densities limited cell extension and migration as well as hindered the deposition of aggrecan and Col II, as confirmed using 3D immunofluorescence and Western blotting. As shown via Masson's trichrome and toluidine blue staining, aggrecan and Col II were evenly distributed in the 5% GelMA hydrogel, indicating that the two ECM components of different molecular weights can be effectively transported in the hydrogel. Combined, our results broaden the potential applications of GelMA in tissue engineering. The GelMA hydrogel allows cells to adhere, grow, and migrate. At the same time, cells encapsulated within the GelMA hydrogel can extend, form connections, and express ECM proteins. Our findings indicate that the 5% GelMA hydrogel is optimal for cell migration and connections in a 3D space and is suitable for supporting NP regeneration and producing ECM.

## 5 CONCLUSION

In this study, we synthesized a photocrosslinked GelMA hydrogel with adjustable mechanical and hydration properties, providing an ideal scaffold for NP regeneration. By changing the ratio of methacrylate, the physical and biological properties of the GelMA hydrogel could be effectively controlled to meet the requirements for NP regeneration. The 5% GelMA hydrogel showed the best performance for cell proliferation, cell morphology, and aggrecan and Col II expression. These data indicate that GelMA hydrogels are a promising scaffold for NP regeneration in the treatment of DDD.

## CONFLICT OF INTEREST

The authors confirm that there are no conflicts of interest.

## AUTHOR CONTRIBUTIONS

**Panpan Xu:** Data curation (equal); Investigation (equal); Methodology (equal); Writing‐original draft (equal). **Jingjing Guan:** Data curation (equal); Methodology (equal); Writing‐original draft (equal). **Yu Chen:** Data curation (equal); Investigation (equal); Methodology (equal); Writing‐original draft (equal). **Hui Xiao:** Data curation (equal); Methodology (equal); Writing‐original draft (equal). **Tianhao Yang:** Data curation (equal); Methodology (equal); Writing‐original draft (equal). **Hengheng Sun:** Data curation (equal); Methodology (equal); Writing‐original draft (equal). **Nan Wu:** Data curation (equal); Methodology (equal); Writing‐original draft (equal). **Changchun Zhang:** Conceptualization (equal); Funding acquisition (equal); Methodology (equal); Writing‐original draft (equal); Writing‐review & editing (equal). **Yingji Mao:** Conceptualization (equal); Data curation (equal); Funding acquisition (equal); Methodology (equal); Writing‐original draft (equal); Writing‐review & editing (equal).

## Data Availability

Data are available on request from the authors.

## References

[1] Juniper M , Le TK , Mladsi D . The epidemiology, economic burden, and pharmacological treatment of chronic low back pain in France, Germany, Italy, Spain and the UK: a literature‐based review. Expert Opin Pharmacother. 2009;10:2581‐2592. 19874246 10.1517/14656560903304063

[2] Pellise F , Balague F , Rajmil L , et al. Prevalence of low back pain and its effect on health‐related quality of life in adolescents. Arch Pediatr Adolesc Med. 2009;163:65‐71. 19124706 10.1001/archpediatrics.2008.512

[3] Martino AD , Vaccaro AR , Lee JY , et al. Nucleus pulposus replacement: basic science and indications for clinical use. Spine. 2005;30:S16‐S22. 16103829 10.1097/01.brs.0000174530.88585.32

[4] Henry N , Clouet J , Le Bideau J , et al. Innovative strategies for intervertebral disc regenerative medicine: from cell therapies to multiscale delivery systems. Biotechnol Adv. 2018;36:281‐294. 29199133 10.1016/j.biotechadv.2017.11.009

[5] Johannessen W , Elliott DM . Effects of degeneration on the biphasic material properties of human nucleus pulposus in confined compression. Spine. 2005;30:E724‐E729. 16371889 10.1097/01.brs.0000192236.92867.15

[6] Huang B , Zhuang Y , Li CQ , et al. Regeneration of the intervertebral disc with nucleus pulposus cell‐seeded collagen II/hyaluronan/chondroitin‐6‐sulfate tri‐copolymer constructs in a rabbit disc degeneration model. Spine (Phila Pa 1976). 2011;36:2252‐2259. 21358466 10.1097/BRS.0b013e318209fd85

[7] Cooper PR , Frempong‐Boadu A . MRI of degenerative disease of the lumbar spine. Magn Reson Q. 1994;10:173‐190. 7811610

[8] Horner HA , Urban JPG . Volvo award winner in basic science studies: effect of nutrient supply on the viability of cells from the nucleus pulposus of the intervertebral disc. Spine. 2001;26:2543‐2549. 11725234 10.1097/00007632-200112010-00006

[9] Silva‐Correia J , Gloria A , Oliveira MB , et al. Rheological and mechanical properties of acellular and cell‐laden methacrylated gellan gum hydrogels. J Biomed Mater Res A. 2013;101:3438‐3446. 23568694 10.1002/jbm.a.34650

[10] Curvello R , Raghuwanshi VS , Garnier G . Engineering nanocellulose hydrogels for biomedical applications. Adv Colloid Interface Sci. 2019;267:47‐61. 30884359 10.1016/j.cis.2019.03.002

[11] Chou AI , Nicoll SB . Characterization of photocrosslinked alginate hydrogels for nucleus pulposus cell encapsulation. J Biomed Mater Res A. 2009;91:187‐194. 18785646 10.1002/jbm.a.32191

[12] Sun Z , Luo B , Liu Z , et al. Effect of perfluorotributylamine‐enriched alginate on nucleus pulposus cell: implications for intervertebral disc regeneration. Biomaterials. 2016;82:34‐47. 26741882 10.1016/j.biomaterials.2015.12.013

[13] Cruz MA , Hom WW , DiStefano TJ , et al. Cell‐seeded adhesive biomaterial for repair of annulus fibrosus defects in intervertebral discs. Tissue Eng Part A. 2018;24:187‐198. 29214889 10.1089/ten.tea.2017.0334 PMC5792242

[14] Perka C , Arnold U , Spitzer R‐S , et al. The use of fibrin beads for tissue engineering and subsequential transplantation. Tissue Eng. 2001;7:359‐361. 11429155 10.1089/10763270152044215

[15] Collin EC , Grad S , Zeugolis DI , et al. An injectable vehicle for nucleus pulposus cell‐based therapy. Biomaterials. 2011;32:2862‐2870. 21276612 10.1016/j.biomaterials.2011.01.018

[16] Sakai D , Mochida J , Yamamoto Y , et al. Transplantation of mesenchymal stem cells embedded in Atelocollagen^®^ gel to the intervertebral disc: a potential therapeutic model for disc degeneration. Biomaterials. 2003;24:3531‐3541. 12809782 10.1016/s0142-9612(03)00222-9

[17] Lin HA , Varma DM , Hom WW , et al. Injectable cellulose‐based hydrogels as nucleus pulposus replacements: assessment of in vitro structural stability, ex vivo herniation risk, and in vivo biocompatibility. J Mech Behav Biomed Mater. 2019;96:204‐213. 31054515 10.1016/j.jmbbm.2019.04.021 PMC6562054

[18] Reza AT , Nicoll SB . Characterization of novel photocrosslinked carboxymethylcellulose hydrogels for encapsulation of nucleus pulposus cells. Acta Biomater. 2010;6:179‐186. 19505596 10.1016/j.actbio.2009.06.004

[19] Silva‐Correia J , Miranda‐Goncalves V , Salgado AJ , et al. Angiogenic potential of gellan‐gum‐based hydrogels for application in nucleus pulposus regeneration: in vivo study. Tissue Eng Part A. 2012;18:1203‐1212. 22439824 10.1089/ten.TEA.2011.0632

[20] Tsaryk R , Silva‐Correia J , Oliveira JM , et al. Biological performance of cell‐encapsulated methacrylated gellan gum‐based hydrogels for nucleus pulposus regeneration. J Tissue Eng Regen Med. 2017;11:637‐648. 25370800 10.1002/term.1959

[21] Di Martino A , Sittinger M , Risbud MV . Chitosan: a versatile biopolymer for orthopaedic tissue‐engineering. Biomaterials. 2005;26:5983‐5990. 15894370 10.1016/j.biomaterials.2005.03.016

[22] Roughley P , Hoemann C , DesRosiers E , et al. The potential of chitosan‐based gels containing intervertebral disc cells for nucleus pulposus supplementation. Biomaterials. 2006;27:388‐396. 16125220 10.1016/j.biomaterials.2005.06.037

[23] Smith LJ , Gorth DJ , Showalter BL , et al. In vitro characterization of a stem‐cell‐seeded triple‐interpenetrating‐network hydrogel for functional regeneration of the nucleus pulposus. Tissue Eng Part A. 2014;20:1841‐1849. 24410394 10.1089/ten.tea.2013.0516 PMC4086360

[24] Su WY , Chen YC , Lin FH . Injectable oxidized hyaluronic acid/adipic acid dihydrazide hydrogel for nucleus pulposus regeneration. Acta Biomater. 2010;6:3044‐3055. 20193782 10.1016/j.actbio.2010.02.037

[25] Chan SC , Gantenbein‐Ritter B . Intervertebral disc regeneration or repair with biomaterials and stem cell therapy–feasible or fiction? Swiss Med Wkly. 2012;142:w13598. 22653467 10.4414/smw.2012.13598

[26] Tanaka N , An SH , Lim T‐H , et al. The relationship between disc degeneration and flexibility of the lumbar spine. Spine J. 2001;4:47‐56. 10.1016/s1529-9430(01)00006-7 14588368

[27] Nichol JW , Koshy ST , Bae H , et al. Cell‐laden microengineered gelatin methacrylate hydrogels. Biomaterials. 2010;31:5536‐5544. 20417964 10.1016/j.biomaterials.2010.03.064 PMC2878615

[28] Bahney CS , Lujan TJ , Hsu CW , et al. Visible light photoinitiation of mesenchymal stem cell‐laden bioresponsive hydrogels. Eur Cell Mater. 2011;22:43‐55; discussion 55. 21761391 10.22203/ecm.v022a04 PMC5050040

[29] Chen YC , Lin RZ , Qi H , et al. Functional human vascular network generated in photocrosslinkable gelatin methacrylate hydrogels. Adv Funct Mater. 2012;22:2027‐2039. 22907987 10.1002/adfm.201101662 PMC3422083

[30] Wang LS , Chung JE , Chan PP , et al. Injectable biodegradable hydrogels with tunable mechanical properties for the stimulation of neurogenesic differentiation of human mesenchymal stem cells in 3D culture. Biomaterials. 2010;31:1148‐1157. 19892395 10.1016/j.biomaterials.2009.10.042

[31] Xin T , Gu Y , Cheng R , et al. Inorganic strengthened hydrogel membrane as regenerative periosteum. ACS Appl Mater Interfaces. 2017;9:41168‐41180. 29144723 10.1021/acsami.7b13167

[32] Liu C , Zhou Y , Sun M , et al. Light‐induced cell alignment and harvest for anisotropic cell sheet technology. ACS Appl Mater Interfaces. 2017;9:36513‐36524. 28984126 10.1021/acsami.7b07202

[33] Zhang X , Li J , Ye P , et al. Coculture of mesenchymal stem cells and endothelial cells enhances host tissue integration and epidermis maturation through AKT activation in gelatin methacryloyl hydrogel‐based skin model. Acta Biomater. 2017;59:317‐326. 28684336 10.1016/j.actbio.2017.07.001

[34] Fan L , Liu C , Chen X , et al. Directing induced pluripotent stem cell derived neural stem cell fate with a three‐dimensional biomimetic hydrogel for spinal cord injury repair. ACS Appl Mater Interfaces. 2018;10:17742‐17755. 29733569 10.1021/acsami.8b05293

[35] Li P , Gan Y , Wang H , et al. Role of the ERK1/2 pathway in osmolarity effects on nucleus pulposus cell apoptosis in a disc perfusion culture. J Orthop Res. 2017;35:86‐92. 27035885 10.1002/jor.23249

[36] Li P , Gan Y , Xu Y , et al. Osmolarity affects matrix synthesis in the nucleus pulposus associated with the involvement of MAPK pathways: a study of ex vivo disc organ culture system. J Orthop Res. 2016;34:1092‐1100. 26576043 10.1002/jor.23106

[37] Iatridis JC , Nicoll SB , Michalek AJ , et al. Role of biomechanics in intervertebral disc degeneration and regenerative therapies: what needs repairing in the disc and what are promising biomaterials for its repair? Spine J. 2013;13:243‐262. 23369494 10.1016/j.spinee.2012.12.002 PMC3612376

[38] Boran G , Regenstein JM . Optimization of gelatin extraction from silver carp skin. J Food Sci. 2009;74:E432‐441. 19799664 10.1111/j.1750-3841.2009.01328.x

[39] Lee SJ , Yhee JY , Kim SH , et al. Biocompatible gelatin nanoparticles for tumor‐targeted delivery of polymerized siRNA in tumor‐bearing mice. J Control Release. 2013;172:358‐366. 24036198 10.1016/j.jconrel.2013.09.002

[40] Li DX , Oh YK , Lim SJ , et al. Novel gelatin microcapsule with bioavailability enhancement of ibuprofen using spray‐drying technique. Int J Pharm. 2008;355:277‐284. 18243606 10.1016/j.ijpharm.2007.12.020

[41] Chen C , Tang J , Gu Y , et al. Bioinspired hydrogel electrospun fibers for spinal cord regeneration. Adv Func Mater. 2019;29:1806899.

[42] Dreesmann L , Ahlers M , Schlosshauer B . The pro‐angiogenic characteristics of a cross‐linked gelatin matrix. Biomaterials. 2007;28:5536‐5543. 17889331 10.1016/j.biomaterials.2007.08.040

[43] Kimura Y , Ozeki M , Inamoto T , et al. Adipose tissue engineering based on human preadipocytes combined with gelatin microspheres containing basic fibroblast growth factor. Biomaterials. 2003;24:2513‐2521. 12695078 10.1016/s0142-9612(03)00049-8

[44] Raeber GP , Lutolf MP , Hubbell JA . Molecularly engineered PEG hydrogels: a novel model system for proteolytically mediated cell migration. Biophys J. 2005;89:1374‐1388. 15923238 10.1529/biophysj.104.050682 PMC1366622

[45] Lutolf MP , Lauer‐Fields JL , Schmoekel HG , et al. Synthetic matrix metalloproteinase‐sensitive hydrogels for the conduction of tissue regeneration: engineering cell‐invasion characteristics. Proc Natl Acad Sci U S A. 2003;100:5413‐5418. 12686696 10.1073/pnas.0737381100 PMC154359

[46] Zhao X , Lang Q , Yildirimer L , et al. Photocrosslinkable gelatin hydrogel for epidermal tissue engineering. Adv Healthc Mater. 2016;5:108‐118. 25880725 10.1002/adhm.201500005 PMC4608855

[47] Zhou P , Xu P , Guan J , et al. Promoting 3D neuronal differentiation in hydrogel for spinal cord regeneration. Colloids Surf B Biointerfaces. 2020;194:111214. 32599502 10.1016/j.colsurfb.2020.111214

[48] Iatridis JC , Weidenbaum M , Setton LA , et al. Is the nucleus pulposus a solid or a fluid? Mechanical behaviors of the nucleus pulposus of the human intervertebral disc. Spine. 1996;21:1174‐1184. 8727192 10.1097/00007632-199605150-00009

[49] Mann BK , West JL . Cell adhesion peptides alter smooth muscle cell adhesion, proliferation, migration, and matrix protein synthesis on modified surfaces and in polymer scaffolds. J Biomed Mater Res. 2002;60:86‐93. 11835163 10.1002/jbm.10042

[50] Brigham MD , Eng M , Bick A , et al. Mechanically robust and bioadhesive collagen and photocrosslinkable hyaluronic acid semi interpenetrating networks. Tissue Eng Part A. 2009;15:1645‐1653. 19105604 10.1089/ten.tea.2008.0441 PMC2709163

[51] Gilchrist CL , Darling EM , Chen J , et al. Extracellular matrix ligand and stiffness modulate immature nucleus pulposus cell‐cell interactions. PLoS One. 2011;6:e27170. 22087260 10.1371/journal.pone.0027170 PMC3210142

[52] Hunter CJ , Matyas JR , Duncan NA . The functional significance of cell clusters in the notochordal nucleus pulposus. Spine. 2004;29:1099‐1104. 15131437 10.1097/00007632-200405150-00010

[53] Lin HA , Gupta MS , Varma DM , et al. Lower crosslinking density enhances functional nucleus pulposus‐like matrix elaboration by human mesenchymal stem cells in carboxymethylcellulose hydrogels. J Biomed Mater Res A. 2016;104:165‐177. 26256108 10.1002/jbm.a.35552

